# Gene(s) and individual feeding behavior: Exploring eco‐evolutionary dynamics underlying left‐right asymmetry in the scale‐eating cichlid fish *Perissodus microlepis*

**DOI:** 10.1002/ece3.4070

**Published:** 2018-05-08

**Authors:** Francesca Raffini, Carmelo Fruciano, Axel Meyer

**Affiliations:** ^1^ Lehrstuhl für Zoologie und Evolutionsbiologie Department of Biology University of Konstanz Konstanz Germany; ^2^ International Max Planck Research School (IMPRS) for Organismal Biology University of Konstanz Konstanz Germany; ^3^ School of Earth, Environmental & Biological Sciences Queensland University of Technology Brisbane QLD Australia; ^4^ Radcliffe Institute for Advanced Study Harvard University Cambridge Massachusetts; ^5^ Institut de biologie de l’Ecole normale supérieure (IBENS) Ecole normale supérieure, CNRS, INSERM PSL Université, Paris France

**Keywords:** asymmetry, candidate SNP, cichlid fish, eco‐evolutionary dynamics, frequency‐dependent selection, niche specialization

## Abstract

The scale‐eating cichlid fish *Perissodus microlepis* is a textbook example of bilateral asymmetry due to its left or right‐bending heads and of negative frequency‐dependent selection, which is proposed to maintain this stable polymorphism. The mechanisms that underlie this asymmetry remain elusive. Several studies had initially postulated a simple genetic basis for this trait, but this explanation has been questioned, particularly by reports observing a unimodal distribution of mouth shapes. We hypothesize that this unimodal distribution might be due to a combination of genetic and phenotypically plastic components. Here, we expanded on previous work by investigating a formerly identified candidate SNP associated to mouth laterality, documenting inter‐individual variation in feeding preference using stable isotope analyses, and testing their association with mouth asymmetry. Our results suggest that this polymorphism is influenced by both a polygenic basis and inter‐individual non‐genetic variation, possibly due to feeding experience, individual specialization, and intraspecific competition. We introduce a hypothesis potentially explaining the simultaneous maintenance of left, right, asymmetric and symmetric mouth phenotypes due to the interaction between diverse eco‐evolutionary dynamics including niche construction and balancing selection. Future studies will have to further tease apart the relative contribution of genetic and environmental factors and their interactions in an integrated fashion.

## 1 INTRODUCTION

**Figure 1:**
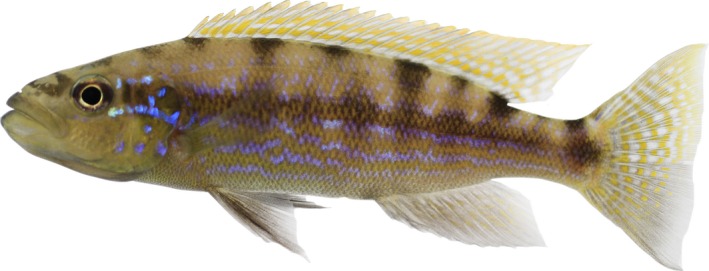
The scale‐eating cichlid fish *Perissodus microlepis*

Stable polymorphisms such as conspicuous asymmetry (departure from symmetry in morphology) or handedness (lateralized behavior) have repeatedly emerged in both vertebrates and invertebrates (Ludwig, 1932; Palmer, 2004, 2009, 2016; e.g., Hori, 1993; Kurvers et al., 2017; Lucky, Ihara, Yamaoka, & Hori, 2012; Matsui, Takeuchi, & Hori, 2013; Takeuchi & Hori, 2008; Tobo, Takeuchi, & Hori, 2012). Yet, the evolutionary forces and the underlying genetic and developmental mechanisms underpinning most of these stable asymmetries (i.e., excluding fluctuating asymmetries) remain unclear (Palmer, 2016; Uzoigwe, 2013). Bilateral asymmetries, where left or right individuals differ from typically bilateral symmetrical specimens, therefore provide fascinating models to study the evolution and the relative contribution of genes and non‐genetic factors to phenotypes (Palmer, 2016). An outstanding model for this research is the scale‐eating cichlid fish *Perissodus microlepis* (Figure 1) from Lake Tanganyika (Africa), where individuals with left (“L”) and right (“R”) bending mouths are found in sympatry in approximately equal frequencies (Hori, 1993; Kusche, Lee, & Meyer, 2012). Its asymmetric mouth is associated with its lateralized foraging behavior: L fish preferentially feed on the scales of the right side of their victim fish, while R individuals bite the scales off from the left side (Hori, 1993; Lee, Kusche, & Meyer, 2012; Takeuchi, Hori, & Oda, 2012; Takeuchi, Hori, Tada, & Oda, 2016; Van Dooren, van Goor, & van Putten, 2010). This polymorphism is thought to be maintained through negative frequency‐dependent selection, where the rare morph gains a selective advantage over the abundant one due to the potential prey's guarding behavior (Hori, 1993; Nakajima, Matsuda, & Hori, 2004). The suggested role of balancing selection made *P. microlepis* also a textbook example of extreme adaptation (Futuyma, 2009; Hori, 1993; Lee, Heim, & Meyer, 2015). However, the developmental and genetic mechanisms that determine this polymorphism remain poorly understood. Three main explanations have been proposed to date: strictly genetic (due to a single Mendelian locus, Hori, 1993; Hori, Ochi, & Kohda, 2007; Stewart & Albertson, 2010), totally or partially random (Palmer, 2004, 2010), and multifactorial (Lee et al., 2015; Palmer, 2010; Raffini, Fruciano, Franchini, & Meyer, 2017; Stewart & Albertson, 2010; Van Dooren et al., 2010) determination of mouth asymmetry. The first two models are hard to reconcile with multiple findings that emerged in the last decade: a) unimodal distribution of mouth shape in both adults and larvae (Kusche et al., 2012; Lee et al., 2015; Stewart & Albertson, 2010; Van Dooren et al., 2010), which is not consistent with a single Mendelian locus; b) parents‐offspring frequencies that do not match expectations for a trait controlled by a single simple locus or a partially random determination of the direction of laterality as seen in mice's internal asymmetry (Lee et al., 2015; Palmer, 2010); c) a significant heritability or single‐nucleotide polymorphisms (SNPs) significantly associated with laterality (Lee et al., 2015; Raffini et al., 2017), which are incompatible with a purely random basis of mouth asymmetry; d) evidence for trait plasticity (Kusche et al., 2012; Lee et al., 2012; Takeuchi et al., 2016; Van Dooren et al., 2010), which is inconsistent with a strictly genetic basis. Mouth asymmetry in *P. microlepis* then is a complex trait (third model, Lee et al., 2015; Palmer, 2010; Raffini et al., 2017; Stewart & Albertson, 2010; Van Dooren et al., 2010), whose variation is most likely due to a polygenic basis and non‐genetic factors (Kusche et al., 2012; Lee et al., 2012; Raffini et al., 2017; Stewart & Albertson, 2010; Takeuchi & Oda, 2017; Takeuchi et al., 2016; Van Dooren et al., 2010). The purpose of this study is to integrate across genetic and environmental factors to further clarify their relative importance.

Several studies suggested that mouth asymmetry in *P. microlepis* has a sizable genetic component (Hori, 1993; Hori et al., 2007; Lee et al., 2015; Palmer, 2010; Raffini et al., 2017; Stewart & Albertson, 2010). This leads to the question: which regions of the genome contain the gene(s) responsible for asymmetry? A microsatellite locus was suggested to be linked to a putative laterality gene (Stewart & Albertson, 2010), but subsequent studies failed to confirm this association (Lee et al., 2010, 2015). More recently, we conducted a genome‐wide study that identified several SNPs potentially related to mouth asymmetry (Raffini et al., 2017). However, the association between these SNPs and the trait could be false positive resulting from factors such as a moderate sample sizes, necessitated by the costs of next‐generation sequencing. Thus, new investigations were needed to validate these candidate loci. In particular, our recent study (Raffini et al., 2017) identified a single SNP by ddRAD sequencing of individuals presenting the most extreme L and R mouth morph (as opposed to multiple loci identified using pools of individuals). In the same study, the position and the gene context of this SNP were ascertained using the genomes of two other African cichlids fish: *Pundamilia nyereri* and *Maylandia zebra*. Our candidate SNP is located near the 5′ end (nucleotide 113) of a 146 bp RAD locus (ID: 56537) placed on a scaffold (50,966 bp) containing three genes and one pseudogene for immunoglobulins. More specifically, this RAD locus is located in the non‐coding region between two of these genes (LOC101464138 and LOC101465275, Raffini et al., 2017). The first aim of this study was to validate this SNP by testing its association with asymmetry using a larger sample size and Sanger sequencing, which has a lower sequencing error. Additionally, while previous studies investigated genomic loci underlying the difference between L and R morphs (i.e., the direction of mouth asymmetry, Hori, 1993; Hori et al., 2007; Lee et al., 2015; Palmer, 2010; Raffini et al., 2017; Stewart & Albertson, 2010), here, for the first time we extend this work by analyzing the association between the candidate locus and mouth bending angle, a more precise and continuous quantification of asymmetry.

Clearly, adaptive phenotypes do not necessarily have a strictly genetic basis, but they can also vary due to phenotypic plasticity (Bradshaw, 1965; reviewed in Pfennig et al., 2010; Pigliucci & Rausher, 2007; Schlichting, 2004; Schneider & Meyer, 2017; West‐Eberhard, 2003; Whitman & Agrawal, 2009). This might be the case for *P. microlepis’* mouth asymmetry, whose heritability estimates indicated also a relatively large environmental component (around 80%, Lee et al., 2015). Several studies analyzed the non‐genetic basis of this trait, suggesting that feeding experience plays an important role, particularly for the amount of asymmetry (Kusche et al., 2012; Lee et al., 2012; Nshombo, Yanagisawa, & Nagoshi, 1985; Takahashi, Watanabe, Nishida, & Hori, 2007; Takeuchi & Oda, 2017; Takeuchi et al., 2016; Van Dooren et al., 2010). They also showed that juveniles and adults primarily feed on scales, but also on alternative prey such as algae, copepods, atyid shrimps, insects’ larvae, benthic animals, and food collected from the substrate (Nshombo et al., 1985; Takeuchi et al., 2016). Interestingly, more scales have been found in the stomachs of individuals with more pronounced degree of mouth asymmetry—suggesting that laterality might be beneficial for scale eating (Takeuchi et al., 2016). Ecological theory predicts that, especially within species, a generalist (in our case, less asymmetric fish feeding on fewer scales and more other items) would be outperformed by a specialist (more asymmetric fish relying more on scales) in the presence of ecological conditions favorable to the specialist (e.g., the specialist's resources are not limited), possibly resulting in a selection for more extreme phenotypes (MacArthur & Levins, 1964; Morris, 1996). However, two specialists and a generalist can also stably co‐exist (e.g., Abrams, 2006; Bono, Gensel, Pfennig, & Burch, 2015; Büchi & Vuilleumier, 2014; Egas, Dieckmann, & Sabelis, 2004; Eloranta, Knudsen, & Amundsen, 2013; Rueffler, Van Dooren, & Metz, 2006). Earlier analyses of feeding behavior in *P. microlepis* mainly concentrated on differences between age classes (Nshombo et al., 1985; Takeuchi et al., 2016), but overlooked potential partitioning into generalists and specialists at the same developmental stage. Such ecological differentiation could emerge due to factors such as diet preference, or stochastic inter‐individual variation in prey items encountered and their proportions. These, in turn, might be one of the causes for variation in the level of asymmetry between individuals. The jaw apparatus of cichlids is famously plastic and adaptable (e.g., Galis & Metz, 1998; Greenwood, 1965; Huysseune, Sire, & Meunier, 1994; Meyer, 1987; Muschick, Barluenga, Salzburger, & Meyer, 2011) and, specifically, in *P. microlepis* is influenced by feeding experience (Lee et al., 2012; Takeuchi et al., 2016; Van Dooren et al., 2010). Thus, the observed unimodal distribution of mouth shapes could be the result of a combination of polygenetic basis and inter‐individual non‐genetic variation due to random (e.g., stochastic variation in feeding behavior) and non‐random (e.g., prey preference) factors. The second goal of this study is, then, to explore individual‐level variation in feeding behavior (niche partitioning) and test its association with the degree of mouth asymmetry.

Previous studies on *P. microlepis’* feeding behavior have used stomach content analyses (Nshombo et al., 1985; Takeuchi et al., 2016). This approach has the advantage of directly measuring what has been ingested. However, this technique has also various limitations, the most critical of which are, in our case, that it reflects only feeding during short periods immediately before capture (Menzel, 1959; Michener & Schell, 1994; Vander Zanden & Rasmussen, 1999). Morphological changes associated with feeding plasticity involve bone and soft‐tissues remodeling, which require some months to show plastic divergence in fish (e.g., Gunter et al., 2013; Schneider, Li, Meyer, & Gunter, 2014; Wimberger, 1991; Witten & Huysseune, 2009). Hence, differences in diet (such as feeding on more scales or alternative prey) should not be sporadic to induce a plastic change and produce different phenotypes (such as more or less asymmetrical mouths) through plasticity. We, therefore, focused on the investigation of longer‐term (months/years) feeding habits through the analysis of stable isotope markers, which provide time‐integrated information of the individual diet. Stable isotope analysis is the identification of the distribution of chemical isotopes within organisms’ tissues. It has been successfully used to address many issues in ecology, supplementing measures from stomach contents (reviewed in Araújo, Bolnick, & Layman, 2011; Bearhop, Adams, Waldron, Fuller, & MacLeod, 2004; examples from cichlid fish: Ford et al., 2016; Hata, Shibata, Omori, Kohda, & Hori, 2015; Kavembe, Kautt, Machado‐Schiaffino, & Meyer, 2016; Malinsky et al., 2015), and it is particularly useful to analyze diet differences among individuals, as variation in feeding behavior is reflected in their isotopic differences (Araújo, Bolnick, Machado, Giaretta, & Dos Reis, 2007; Fry et al., 1999). Trophic studies typically use the naturally occurring carbon (δ^13^C) and nitrogen (δ^15^N) stable isotope. The first provides information on the original source of carbon to the food web. In lacustrine animals, planktonic primary producers are depleted in δ^13^C compared to benthic primary producers and their respective predators (DeNiro & Epstein, 1978; Hecky & Hesslein, 1995). The second isotope, δ^15^N, gives insight into an organism's trophic position, as it consistently increases with rising trophic level as the lighter nitrogen isotope is preferentially excreted (Cabana & Rasmussen, 1994).

In this study, we analyzed inter‐individual variation both at the genetic level at a candidate genomic locus and in stable isotopes composition, pursuing a much‐needed integrative perspective unifying aspects of genetic and environmental determination. For the first time, our findings confirmed the association between a candidate locus and mouth asymmetry, documented established individual feeding specialization related to mouth phenotype among fish at the same developmental stage, provided further evidence for a quantitative basis of asymmetry, and proposed a mechanism that reconciles previously contrasting observations and comprehensively explains the origin and maintenance of the whole (direction and degree) mouth polymorphism.

## 2 MATERIALS AND METHODS

**Figure 2:**
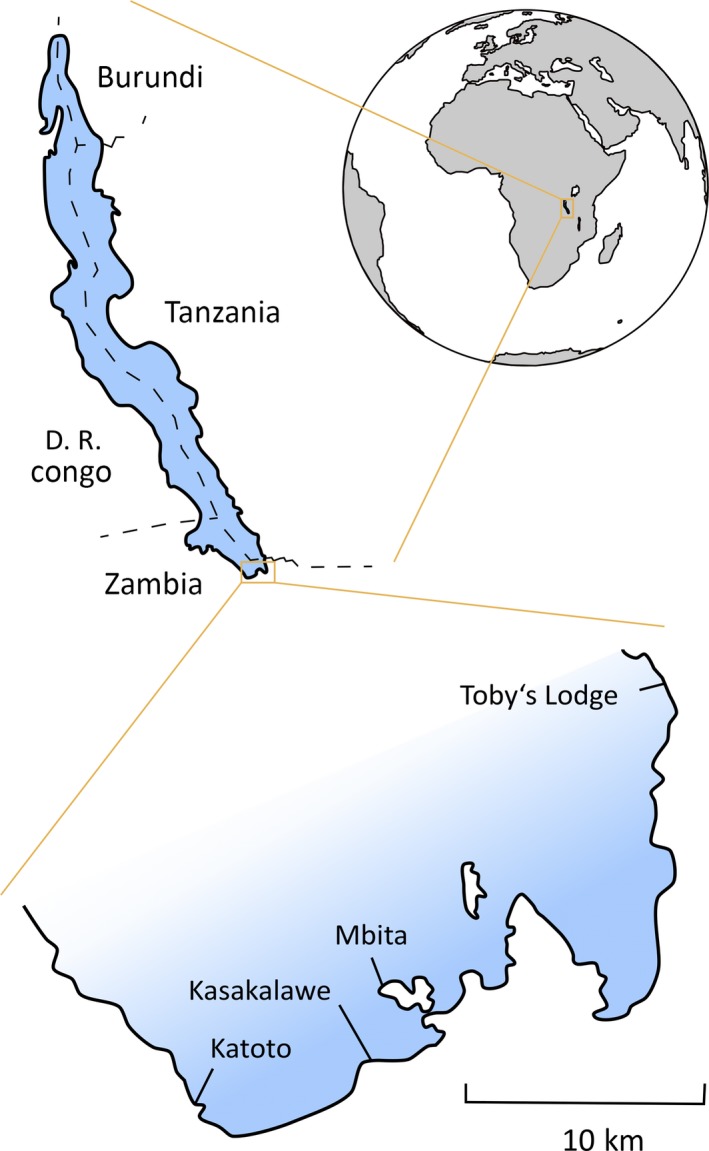
Lake Tanganyika sampling locations in Zambia (Africa). Countries are reported in capital letters, sampling sites with regular front. National borders are indicated with dashed lines

A total of 239 adult fish were collected in April 2010 from four locations on the Zambian coast of Lake Tanganyika (Figure 2), and preserved in ethanol (Kusche et al., 2012; Raffini et al., 2017), according to the study permit (G.R. no: 2077761) issued from the Department of Immigration of the Republic of Zambia (Kusche et al., 2012). All methods were carried out in accordance with relevant guidelines and regulations.

The degree of mouth asymmetry was measured as reported in Raffini et al. (2017). Briefly, using photographs of the top of the head, we measured the mouth‐bending angle of each individual. Three points corresponding to the most anterior part of the eye sockets and the tip of the snout were digitized on photographs. From the coordinates of these points, the angles at each of the eye sockets were computed. The degree of asymmetry was measured as the difference between angles at the left and right eye. Positive values indicate left‐bending (“left”) individuals, whereas negative results are indicative of right‐bending (“right”) fish.

Fish age has been frequently estimated analyzing calcified structures such as scales or otoliths, but it has often proven difficult in tropical fishes, as they do not experience marked seasonal variations in environmental conditions or reproductive activity (Longhurst & Pauly, 1987). A preliminary survey of scale rings in *P. microlepis* suggested that this species is not an exception. Therefore, we used body size as a proxy for age (Petersen, 1894), as previously done also in this species (Takeuchi et al., 2016). Individual body sizes were retrieved from standardized photographs of the body in lateral view as body centroid size (Raffini, Fruciano, & Meyer, in press), taking averages of repeated measurements to reduce the measurement error (Fruciano, 2016; see online Appendix S1 for details).

### 2.1 SNP validation

A previous study (Raffini et al., 2017) identified one SNP probably related to the differentiation between the L and R morph through ddRAD sequencing. This locus is located in a non‐coding region containing immunoglobulin genes and has two alternative alleles: G, associated to the right morph, and A, related to the left morph (Raffini et al., 2017).

Here, this candidate SNP 56537‐113 was amplified and sequenced. Genomic DNA was extracted from finclips and amplified using specific PCR primers we designed for this study (Appendix S1). 168 successfully amplified PCR products were postprocessed and sequenced on a 3130xl ABI sequencer and then aligned using the ClustalW algorithm with default settings in MEGA v. 7.0 (Kumar, Stecher, & Tamura, 2016; details in Appendix S1). The consensus sequence of the locus 56537 was incorporated in the alignment to localize the candidate SNP (56537‐113), and each individual was genotyped at this position. Deviation from the Hardy–Weinberg Equilibrium was tested in R v. 3.3.1 (R Core Team, 2016) using the package *genetics* (Warnes, Gorjanc, Leisch, & Man, 2013).

To test for the association between the SNP genotype and mouth bending angle, we conducted statistical analyses in R. A model selection approach was used to select the quantitative genetic model that best characterized this SNP (Appendix S1). The genotype scoring corresponding to the best fitting model was then used in ANOVA and PERMANOVA analyses (Anderson, 2001; Excoffier, Smouse, & Quattro, 1992; Fisher, 1919; Legendre & Anderson, 1999; Warton, Wright, & Wang, 2012; *adonis* function, R package *vegan,* Oksanen et al., 2016, using Euclidean distances and 999 permutations).

The fish were sampled at four different locations (Figures 2; Table S2), and geographic structure could be a confounding factor (Koblmüller et al., 2009; Raffini et al., 2017). Therefore, ANOVA and PERMANOVA analyses were repeated including also sampling location as explanatory variable.

### 2.2 Stable isotope analysis

We selected the 34 adult individuals with the most extreme mouth bending angle (“asymmetric” phenotype, 17 L and 17 R), and the 34 most symmetric (“symmetric” phenotype) samples (Table S3). Similar or lower sample sizes had previously been successful in detecting feeding differences in other fish species (e.g., Ford et al., 2016; Kavembe et al., 2016; Manousaki et al., 2013) and the selection of the most extreme specimens contributes to maximize the power of finding differences between groups. The diet during the period over which a tissue is synthesized determines the stable isotope signatures of the tissue. Diverse tissues have different isotope turnover rates, and thus integrate dietary information over different time windows (Pinnegar & Polunin, 1999; Tieszen, Boutton, Tesdahl, & Slade, 1983). Therefore, we processed and analyzed both white muscle and bone tissues extracted from dorsal musculature and abdominal vertebrae of each fish using standard procedures (Appendix S1), to gain a more comprehensive and less ambiguous data on both individual intermediate (months, muscle) and long‐term (years, bone) feeding habit, respectively (Gaston & Suthers, 2004; MacNeil, Drouillard, & Fisk, 2006; Tieszen et al., 1983).

The relationship between stable isotopes values and mouth phenotype (asymmetric/symmetric) was statistically analyzed in R. Prior to these analyses, we performed the Shapiro–Wilk test (Shapiro & Wilk, 1965) for normal distribution. One dataset, muscle δ^13^C, did not follow a normal distribution, similarly to what observed in other cichlids (e.g., Elmer, Lehtonen, Kautt, Harrod, & Meyer, 2010); thus, we used both parametric and non‐parametric statistics. Univariate (ANCOVA, one for each stable isotope and tissue) and multivariate analyses of covariance (MANCOVA, one for each tissue) were performed using δ^13^C and/or δ^15^N (response variables) and the mouth phenotype (asymmetric/symmetric, predictor variable). To allow for heteroscedasticity, we also used generalized least squares models (Aitken, 1935; R functions *gls* and *varIdent*, library *nlme* v. 3.1‐128, Pinheiro, Bates, DebRoy, & Sarkar, 2016). In particular, we fit models with both constant (equivalent to the general linear models described above) and different (structured, one for each phenotype, asymmetric/symmetric) variance. As sampling location and body size (Table S3) can contribute to variation in stable isotopes values, we also included them in our models, both with and without interaction between variables. These different models were compared using a model selection approach (AICc, *AICctab* and likelihood ratio test, Appendix S1, Neyman & Pearson, 1933; R function *anova,* Chambers, Freeny, & Heiberger, 1992).

Variance in stable isotopes was used as a measure of niche width (Bearhop et al., 2004). Specifically, we tested for homogeneity of variance between the two mouth phenotypes (asymmetric/symmetric) for each isotope dataset using the variance ratio test (*F*‐test, as in Bearhopet al., 2004), and Levene's test (more robust to departures from normality, Levene, 1960; R package *car,* Fox & Weisberg, 2011). To take into account the effect of sampling location and size, we first fit linear models using location and size as predictors, and then tested for the equality of variances of residuals (all normally distributed; Shapiro–Wilk test *p*‐value > .05). This analysis was performed to test if the asymmetric (pooling individuals with extreme L and R morph) fish have a more specialized diet when compared to the symmetric individuals. If this were true, it would lend support to the idea of a significant relationship between individual specialization and the level of asymmetry.

## 3 RESULTS

### 3.1 SNP validation

To verify a previously identified SNP and its relationship with the degree of mouth asymmetry, we explored the association between the candidate SNP 56537‐113 and mouth bending angle. 168 samples were successfully genotyped at this locus (Table S2). These included 22 individuals that were already sequenced with ddRAD in a previous study (Raffini et al., 2017); 21 of 22 samples matched between the ddRAD and PCR genotyping. The single mismatch is likely due to sequencing error, which is notoriously lower in Sanger sequencing (Shendure & Ji, 2008). The following variants were observed: homozygous for A (AA), homozygous for G (GG), or heterozygous (AG/GA), without the presence of other nucleotides. This locus significantly deviates from the Hardy–Weinberg Equilibrium (number of individuals with genotype GG = 84, AA = 44, AG/GA = 40, exact test for Hardy–Weinberg Equilibrium *p* = 1.346e^−10^).

**Figure 3:**
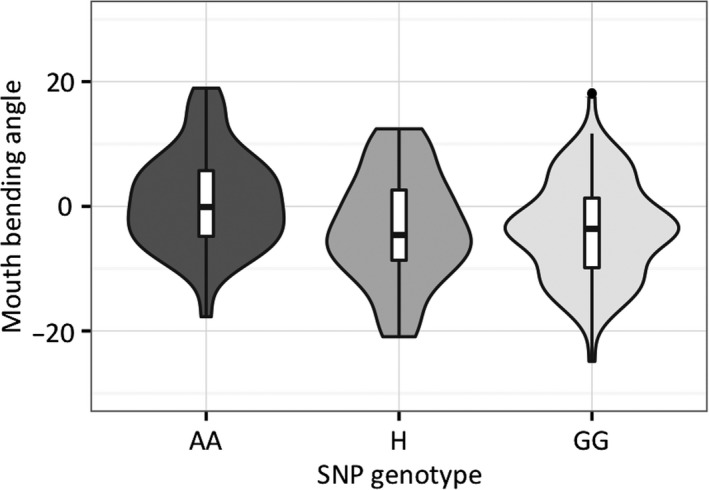
Violin plots of mouth bending angle by genotype at the SNP 56537‐113 locus. The boxplots show the group median (black horizontal lines), first and third quartiles (the 25th and 75th percentiles; hinges), and 95% confidence interval of median (notches). Three individuals from the Crocodile sampling site were excluded from analysis (see main text). H, heterozygous (genotype AG or GA)

The three quantitative genetic models (A dominant, G dominant, totally additive) were all statistically significant for our genetic data; the G dominant model was the one that best characterizes this SNP (Table S4). The results were all concordant in indicating a statistically significant association between the mouth bending angle and the candidate SNP, and that variation at this locus accounts for about 6% of the trait variation (Figure 3; ANOVA *F*_1,166_ = 10.66, *p *=* *.001329, *R*‐squared = .06035; PERMANOVA *F*_1,166_ = 10.662, *p *=* *.002, *R*‐squared = .06035). This relationship remained significant when sampling sites (a potential confounding factor) were included in the model (ANOVA mouth angle: *F*_1,160_ = 10.458, *p *=* *.00148; locations: *F*_3,160_ = 0.753, *p *=* *.52219; multiple *R*‐squared = .07672; PERMANOVA mouth angle: *F*_1,160_ = 10.4584, *p *=* *.001, *R*‐squared = .06035; locations: *F*_3,160_ = 0.7529, *p *=* *.539, *R*‐squared = .01303; multiple *R*‐squared = .07338; interactions between mouth angle and locations always *p *>* *.05), that, together with our SNP, explains about 7% of the phenotypic variation.

### 3.2 Medium and long‐term diet analysis

Variation in individual feeding behavior and its association with mouth phenotypes (asymmetric/symmetric, two groups created by selecting the specimens with the highest and lowest mouth bending angle respectively) was investigated through stable isotope analysis of muscle (medium term) ad bone (long term) tissue (Gaston & Suthers, 2004; MacNeil et al., 2006; Tieszen et al., 1983). Three additional outliers presenting clearly distinct stable isotopes values from the rest of specimens were identified in both the muscle (Figure S5, sample ID: 10781, 10862, 10879) and bone (Figure S6, sample ID: 10862, 10877, 10879) datasets. As this deviation was not linked to any known biological differences, they were removed before any subsequent analyses of stable isotope variation to avoid the inclusion of potential artifacts not linked to natural variation. The sample sizes used for analyses in muscle were 31 (32 for bone) and 34 (33 for bone) for asymmetric and symmetric fish, respectively.

Model selection indicated that including mouth phenotype (asymmetric/symmetric), sampling location and body size (a proxy for age) best explain our data (Table S7), while incorporating a variance heterogeneity (i.e., a specific variance for the symmetric and asymmetric group, respectively) was not always the best‐supported option (Table S8). Interaction terms between mouth phenotype (asymmetric/symmetric) and location and size in both ANOVA and MANCOVA were always not significant. We, therefore, fit the various models using only the main terms and discarded interaction terms.

**Figure 4:**
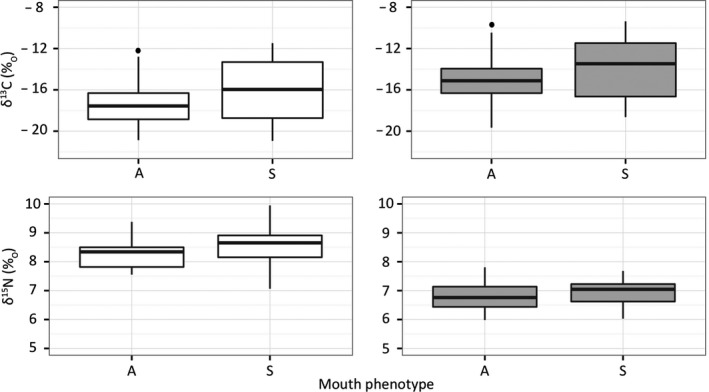
Muscle (white) and bone (gray) carbon and nitrogen isotopic values as a function of the mouth phenotype (asymmetric/symmetric). The boxplots show the group median (black horizontal lines), first and third quartiles (the 25th and 75th percentiles; hinges), and 95% confidence interval of median (notches). Three outliers were excluded from both the muscle and bone datasets (see main text). A, asymmetric phenotype; S, symmetric phenotype

We observed a significant relationship between the carbon stable isotope values and the mouth phenotypes (asymmetric/symmetric) in both medium (muscle) and long (bone) term diet (mean stable isotope values). The asymmetric group was on average depleted (higher negative values) in δ^13^C compared to the symmetric pool. This association was not significant for the nitrogen marker (Figure 4; Table 1; Table S9; Table S10; Table S11; Table S12).

**Table 1:** Univariate ANCOVA results of the stable isotope dataset

Tissue	Stable isotope	Variable	*Df* num, *df* den	*F*‐value	*p*‐value
Muscle	δ^13^C	Mouth phenotype	1, 59	10.855	**.00167**
		Location	3, 59	16.651	**5.934e** ^**−08**^
		Size	1, 59	40.245	**3.451e** ^**−08**^
	δ^15^N	Mouth phenotype	1, 59	3.9582	.05128
		Location	3, 59	3.9393	**.01250**
		Size	1, 59	33.7171	**2.701e** ^**−7**^
Bone	δ^13^C	Mouth phenotype	1, 59	8.5919	**.004798**
		Location	3, 59	15.6956	**1.283e** ^**−07**^
		Size	1, 59	39.1736	**4.790e** ^**−08**^
	δ^15^N	Mouth phenotype	1, 59	1.6414	.2051525
		Location	3, 59	7.4128	**.0002701**
		Size	1, 59	8.2716	**.0055952**

Three outliers were excluded from both the muscle and bone datasets (see main text).

*Df*, degree of freedom.

Significant *p*‐value (< .05) is reported in boldface.

The asymmetric and symmetric phenotypes showed a different niche width (variance in stable isotopes values) in the medium term (muscle). It is larger in the symmetric group, indicative of a broader diet including more various kinds of food, compared to the asymmetric group, which appeared to be more specialized. This difference was not observed for the longer‐term niche width (bone, Table 2; Figure 4; Figure S5; Figure S6). These differences in niche width are clear only when controlling for geographical variation, probably as the contribution to variance due to geography is large enough to obscure the underlying pattern (Table 2).

**Table 2:** Tests of homogeneity of variance for the stable isotope datasets

Dataset	Tissue	Stable isotope	*F*‐test			Levene's test		
			*df*	*F*‐value	*p*‐value	*df*	*F*‐value	*p*‐value
Without correction for location and size	Muscle	δ^13^C	30, 33	0.58406	.1403	1, 63	4.4467	**.03895**
		δ^15^N	30, 33	0.43603	**.0240**	1, 63	2.111	.1512
	Bone	δ^13^C	31, 32	0.6391	.2158	1, 63	3.6558	.06558
		δ^15^N	31, 32	1.3412	.4132	1.63	0.5859	.4469
Residuals corrected for location and size	Muscle	δ^13^C	30, 33	0.4217	**.02085**	1, 63	4.4476	**.03893**
		δ^15^N	30, 33	0.26272	**.003816**	1, 63	6.4373	**.01366**
	Bone	δ^13^C	31, 32	0.52489	.076	1, 63	1.6592	.2024
		δ^15^N	31, 32	1.4341	.3154	1, 63	2.262	.1376

*Df*, degree of freedom.

Significant *p*‐value (< .05) is reported in boldface.

Size and location had a significant effect on the stable isotopes values (Table 1; Table S9; Table S10; Table S11; Table S12), but not on mouth phenotype (asymmetric/symmetric, interactions never significant).

## 4 DISCUSSION

We analyzed the relationship between genetic and ecological (established individual feeding behavior) variation and morphological asymmetry in the scale‐eating fish *Perissodus microlepis*. The results confirm that the candidate SNP 56537‐113 is associated with mouth bending angle, which explains a relatively small amount (6%) of phenotypic variation, and provide further support for the hypothesis that head asymmetry in *P. microlepis* has a complex genetic basis. They also suggest that individual specialization and variation in feeding habits could, in addition to such a complex genetic architecture, contribute to the unimodal distribution of this trait. We introduce a comprehensive hypothesis potentially explaining how these genetic and non‐genetic cues jointly influence the direction and the degree of mouth asymmetry as well as the maintenance of intraspecific variation.

### 4.1 A role of the immune system in mouth asymmetry

Several immunity genes were proposed to potentially underlie *P. microlepis’* mouth asymmetry (Raffini et al., 2017). While previous studies failed to validate a former candidate locus (UNH2101; Lee et al., 2015, 2010; Stewart & Albertson, 2010 but note the use of different methods to estimate asymmetry), here, we confirmed the association between the locus located in a genomic region containing immunoglobulin genes and mouth polymorphism. Specifically, the SNP 56537‐113 is related to mouth asymmetry whether this is expressed as a continuous trait (mouth bending angle, this study) or as a categorical variable (L and R morphs, Raffini et al., 2017).

The candidate SNP showed an excess of homozygotes that could be indicative of inbreeding or strong assortative mating, population structure or selection against heterozygous individuals (Haldane, 1954; Hedrick, 2011; Hernandez & Weir, 1989; Levene, 1949; Wellek, 2004). Previous studies suggested random or disassortative mating as well as negative frequency‐dependent selection in *P. microlepis* (Hori, 1993; Kusche et al., 2012; Lee et al., 2010; Takahashi & Hori, 2008 but see Raffini et al., 2017). Restrictions to gene flow linked to geography have been identified in the distribution range of this species (Koblmüller et al., 2009; Raffini et al., 2017, in press); however, we observed a significant genotype‐phenotype relationship also when controlling for geography (Raffini et al., 2017, this study).

Our results (Raffini et al., 2017; this study) seem to indicate a role of the immune system in mouth asymmetry. The immune system is known to contribute to animal diversification also in the absence of geographical boundaries (e.g., Eizaguirre, Lenz, Traulsen, & Milinski, 2009; Landry, Garant, Duchesne, & Bernatchez, 2001; Malmstrøm et al., 2016; discussed in Raffini et al., 2017). However, the immunoglobulin locus associated with the identified SNP might also have indirect effects on mouth shapes due to functional or physical association to the genomic locus (or loci) for asymmetry (e.g., Lehnert, Pitcher, Devlin, & Heath, 2016; Sacchi et al., 2007). Linkage rather than a direct causal relationship or a polygenetic basis for mouth asymmetry (as discussed in Raffini et al., 2017) could explain the relatively small proportion of mouth phenotypic variation accounted for by this SNP, including when the geographic variation is considered. Alternatively, the location of this SNP in a putatively non‐coding region flanking two genes might suggest that variation in regulatory and not in coding elements may be responsible for phenotypic variation (as seen in e.g., Belting, Shashikant, & Ruddle, 1998; Chan et al., 2010; Cretekos et al., 2008; Guenther, Tasic, Luo, Bedell, & Kingsley, 2014; Guerreiro et al., 2013; Schneider et al., 2014; Shapiro, Marks, Peichel, & Blackman, 2004). Future investigations focusing on immunity‐related processes or loci underlying this polymorphism might further advance our understanding of the genetic architecture of the *P. microlepis* head asymmetry.

### 4.2 Individual feeding specialization is related to the degree of asymmetry

This is the first study to investigate medium and long‐term dietary differences in *P. microlepis*, the presence of niche partitioning among adult samples, and its relationship with mouth asymmetry.

The stable isotope signature means of the most asymmetric fish were significantly different from those of the most symmetric individuals. On one hand, the symmetric group consumed on average a higher proportion of carbon of benthic origin (enriched in δ^13^C according to aquatic food webs, France, 1995; Michener & Schell, 1994; Michener & Kaufman, 2008), compatible with a medium and long‐term diet including more benthic‐associated prey such as the alternative food found in *P. microlepis’* stomach (some copepods, benthic animals, atyid shrimps, ephemeroptera, or trichoptera larvae, etc., Nshombo et al., 1985; Takeuchi et al., 2016). On the other hand, the higher negative values of δ^13^C in asymmetric individuals are congruous with eating more pelagic prey, such as fishes from which *P. microlepis* remove scales. This is in agreement with previous findings based on stomach content analysis, where more scales were ingested by more asymmetric specimens (Takeuchi et al., 2016). Only the δ^13^C, but not the δ^15^N signatures were significantly associated with differentiation between two phenotypes (asymmetric/symmetric). Both groups mainly feed on primary consumers (algae‐eater fishes’ scales or copepods, fishes and insects’ larvae, Nshombo et al., 1985; Takeuchi et al., 2016), resulting in related trophic levels, that are reflected in analogous δ^15^N values, similarly to what was reported for some recently diverged species (e.g., Ford et al., 2016; Malinsky et al., 2015).

The two mouth phenotypes (asymmetric/symmetric) showed a different niche width in the medium term, and it was smaller in asymmetric individuals compared to symmetric ones. This could be interpreted as a hallmark of a more specialized, narrow ecological niche in the more asymmetric fish compared to a more generalist diet in the more symmetrical individuals. On a longer timescale, these two phenotypes did not exhibit significant differences in their variance of isotopic composition. This is likely because bones provide an averaged information about assimilated nutrients over several months/years (Gaston & Suthers, 2004; Tieszen et al., 1983). Thus, if differences in niche width are relatively small (such as in sympatric morphs of the same species), these could be masked when integrated over an extended period of time.

Stable isotope signatures were also influenced by sample location and size. The isotopic baseline is typically affected by several environmental factors (e.g., depth, amount of anthropogenic disturbance, local prey community) that can also vary within lakes at small spatial scales (Casey & Post, 2011; Post, 2002). Another known phenomenon in fish is ontogenetic dietary change, reflected in a change in the isotopic signatures with increasing body size (Jardine, McGeachy, Paton, Savoie, & Cunjak, 2003; Mittelbach & Persson, 1998). In *P. microlepis*, a dietary switch occurs from omnivores to predominantly scale‐eating in juveniles (Nshombo et al., 1985; Takeuchi et al., 2016). Our results showed a strong effect of body size on stable isotope values, which might reflect such a feeding change as well. In fact, our fish were all adults but some of them (e.g., fish of centroid size nine) might have changed feeding more recently than others (e.g., fish of centroid size 16), and, since stable isotope value provide time‐integrated information on diet over the past months/years, we observed a correlation between body size and isotopic signature. Importantly, although location and size had higher impact on the stable isotope values, neither of them were significantly associated with the mouth phenotype (asymmetric/symmetric), hence these influences did not affect our analyses of association between mouth asymmetry and stable isotope signatures.

It has been suggested (Takeuchi et al., 2016) that disruptive selection in *P. microlepis* would favor fish having more asymmetric mouth due to improved scale‐eating efficiency, while symmetric samples would be negatively affected in their growth, survival and reproductive rate. Although a conclusive investigation of this hypothesis would require a detailed analysis of mortality rate, hunting, and reproductive success, current data does not seem to support this scenario. In fact, adult symmetric fish are commonly observed in nature (Kusche et al., 2012; Takeuchi et al., 2016; Van Dooren et al., 2010) as reported also in studies with larger sample sizes from a single location (Takeuchi et al., 2016; Van Dooren et al., 2010). In our study, symmetric specimens showed higher interindividual variation in stable isotopes values. This broader variation in isotope signatures is caused by feeding on a larger variety of food items (Bearhop et al., 2004), possibly in an effort to compensate for lower amount of scales (Takeuchi et al., 2016) with alternative food. Estimates of caloric value obtained in each attack showed that feeding on copepods (alternative prey) is comparable to eating scales (Nshombo et al., 1985). Therefore, there is currently no clear evidence of lower fitness of less asymmetric fish, at least in terms of growth and survival. A mechanism other than disruptive selection *via* scale‐feeding efficiency might be responsible for the maintenance of this trait. And, as even extremely specialized cichlids tend to feed opportunistically, only during the most challenging of ecological times might the selective advantage of specialized morphology become important (e.g., Grant & Grant, 1993). We advocate future studies to explore this hypothesis.

Overall, our results showed that differences in medium and long‐term feeding behavior and diet breadth exist between *P. microlepis* individuals at the same developmental stage that have the most and the least asymmetric heads. Considering the direct (Van Dooren et al., 2010) and indirect (Kusche et al., 2012; Lee et al., 2012; Takeuchi et al., 2016) evidence of an impact of phenotypic plasticity through diet on mouth polymorphism, our results may suggest that individual feeding specialization contribute to influence the degree of mouth asymmetry. Future studies will need to further analyze the influence of non‐genetic factors, particularly feeding behavior, and individual specialization, especially in the context of genetic studies (e.g., gene X environment interactions).

### 4.3 Gene(s) & environment: a concerted effect?

According to these and previous results (Hori, 1993; Kusche et al., 2012; Lee et al., 2012, 2015; Nshombo et al., 1985; Raffini et al., 2017; Takeuchi et al., 2016; Van Dooren et al., 2010), the direction of mouth asymmetry could be under genetic control, while the bending angle is influenced by gene(s) possibly together with environmental factors. A similar complex architecture has been reported in human and other fish handedness and brain lateralization (reviewed in Ocklenburg & Gunturkun, 2012).

The suggestion that both genes and environment contribute to variation in *P. microlepis* head asymmetry has been made before (Kusche et al., 2012; Lee et al., 2015; Stewart & Albertson, 2010; Van Dooren et al., 2010). However, a hypothesis on how they jointly influence this polymorphism was lacking so far. Here, we propose that juveniles, that initially attack both flanks of their prey, may learn during their ontogeny at which side they are more efficient in removing scales depending on their overall mouth‐bending direction (Takeuchi & Oda, 2017; Takeuchi et al., 2016), which is genetically determined (Hori, 1993; Raffini et al., 2017; this study) and whose polymorphism could still possibly be maintained through negative frequency‐dependent selection (Hori, 1993). Thereby, fish become increasingly specialized (handed) in preying on their victim fish preferentially from their “adapted” side (Lee et al., 2012; Takeuchi & Oda, 2017; Takeuchi et al., 2012, 2016). In response to this established behavioral lateralization, morphological asymmetry is amplified through plasticity in those individuals eating more scales, resulting in a positive feedback loop between mouth asymmetry and lateralized behavior (Lee et al., 2012; Palmer, 2010; Stewart & Albertson, 2010; Takeuchi et al., 2016; Van Dooren et al., 2010). Conversely, individuals that are potentially less successful in grazing scales (corresponding to the symmetric ones, as stomach content analysis seems to indirectly suggest, Takeuchi et al., 2016) may learn to compensate this source of nutrients through alternative foods (as possibly indicated by this study) that do not stimulate plastic responses in asymmetry. This hypothesis is particularly supported by more pronounced amounts of asymmetry in adults compared to juveniles, the gradual establishment of lateralized behavior during development that is increasingly positively correlated with mouth asymmetry, the presence of higher number of scales in the stomachs of more asymmetric specimens, and the significant increase of variance in the degree of mouth asymmetry with growth (Takeuchi & Oda, 2017; Takeuchi et al., 2016). *P. microlepis* might be a special case of individualized niche construction, as the fish would fit the particular task of prey acquisition to its morphology.

The existence of differences in diet and/or scales‐hunting success within this species can arise from external factors, such as variability in the types of food found, as well as inter‐ or intra‐specific competition (Bono et al., 2015; Mateus, Ortega, Mendes, & Penha, 2016; Schluter, 1994, 2000, 2001). Alternatively, feeding preference might be innate (e.g., under strong genetic or epigenetic control; e.g., Serobyan et al., 2016), or hunting success may strictly depend on the extent of mouth asymmetry (Takeuchi et al., 2012), that in this study appeared to also have an, at least partial, genetic basis. Following this interpretation, both the direction and the amount of asymmetry are genetically based, generating a unimodal distribution of mouth shapes due to the combined effects of their polygenic nature (Raffini et al., 2017; this study) and plasticity *via* feeding experience as described above. Another factor internal to the organism that could contribute to the observed differences in scale‐eating success is the among‐individual variance in learning ability, a well‐documented phenomenon in fish (Fawcett, Hamblin, & Giraldeau, 2013; Kieffer & Colgan, 1991; Versace, 2015). “Fast learners” could start to successfully attack preys earlier, resulting in a higher success and amount of eaten scales during their life, and thus a more accentuated effect of plasticity on the degree of mouth asymmetry compared to “slow apprentices.”

While the precise mechanisms remain to be clarified, a central role for inter‐individual variation emerges as the key to understand the bases of *P. microlepis* mouth polymorphism, reconciling and unifying the largely genetic and environmental determination models. inter‐individual differences have important evolutionary and ecological effects, and constitute a source of variation upon which natural selection can act (Araújo et al., 2011; Dall, Bell, Bolnick, & Ratnieks, 2012; Nosil, 2012; Schluter, 2000, 2001). It might also suggest a mechanism of sympatric coexistence and maintenance of different mouth phenotypes in *P. microlepis*: more asymmetric fish are specialized predators and provisionally more successful scale predators depending on the morphs (left/right) relative abundance (the classic model of negative frequency‐dependent selection, Hori, 1993), while symmetric specimens are more generalist hunters. Mathematical models suggest that evolutionarily stable coexistence of two specialists and a generalist can arise through immigration or mutation, especially in the presence of strong resources temporal variability or consumer‐resources cycles and adaptive foraging behavior (Abrams, 2006; Egas et al., 2004; Rueffler et al., 2006). Then, these factors might contribute to the long‐term maintenance of the trophic polymorphism observed in *P. microlepis*. To our knowledge, this is the first hypothesis introducing a process that can simultaneously explain the presence of left, right, symmetric, and asymmetric fish due to the interaction between different evolutionary and ecological dynamics and response strategies.

This study clarified the relative importance of genetic and environmental factors affecting mouth asymmetry in *P. microlepis*. Our results add to the growing support for a quantitative nature of this trait, confirm a previously identified genomic region as harboring at least one of the loci responsible for it, and emphasize the importance of considering both genetic and external triggers. For the first time, we propose that individuals are partitioning resources (niche specialization/construction) according to their mouth phenotype, which is partly genetically determined, allowing the coexistence and maintenance of different morphs. Importantly, our study highlights the promise of considering inter‐individual variation when aiming to understand how this polymorphism is produced and maintained, and how an integrative view can help reconcile previously distinct observations.

## CONFLICT OF INTEREST

None declared.

## AUTHOR CONTRIBUTIONS

FR, CF, and AM designed the study. Morphological data were collected by FR and analyzed by CF. Stable isotopes samples were preprocessed by FR and analyzed in the Limnological Institute of the University of Konstanz. Genetic data was generated by FR. Raw data were analyzed by FR and CF. FR drafted the manuscript. All authors edited and agreed to the manuscript.

## DATA AVAILABILITY

The datasets generated during this study are included in this published article and its Supplementary Information (Table S1 and S2).
