# Supplementary material for: Gene(s) and individual feeding behavior: Exploring eco‐evolutionary dynamics underlying left‐right asymmetry in the scale‐eating cichlid fish Perissodus microlepis
Source: Ecol Evol. 2018 May 8;8(11):5495–507. doi: 10.1002/ece3.4070 (PMC6010907; doi:10.1002/ece3.4070)
Supplement: Supplementary file 1 [file ECE3-8-5495-s001.pdf]

Supplementary Information

Appendix S1

Measurement of individual body size. We used body size as a proxy for age (Petersen, 1894), as previously done in this species (Takeuchi *et al.*, 2016). Using photographs of both the left and right side of each individual, we digitized a set of 33 points (landmarks, semi-landmarks and helper points; these were used in a study of body shape currently in revision, Raffini *et al.*, in press). To reduce the measurement error, we obtained repeated measurements of centroid size (Fruciano, 2016). In particular, we obtained two pictures per side and digitized two times per picture, totaling eight landmark configurations per fish. We then computed centroid size for each of these configurations and then averaged them by individual (Raffini *et al.*, in press). These average centroid sizes were then used in all subsequent analyses as estimates of body size.

Amplification and sequencing of the candidate locus 56537. Genomic DNA was extracted from finclips using the ZR Genomic DNATM-Tissue MiniPrep kit (Zymo Research). To develop specific PCR primers, first the consensus sequence of the locus 56537 was retrieved from the ddRAD dataset (Raffini *et al.*, 2017). Since the SNP was located close to the end of this sequence rendering it difficult to design reliable primers, it was aligned against the available cichlid reference genomes (Brawand *et al.*, 2014) in Blastn v. 2.2.30 (Altschul *et al.*, 1997) with default settings. Using the resulting cichlid alignment and Primer3Plus (Untergasser *et al.*, 2007), we designed primers in the conserved regions (reverse: 5'TTCCTGTGCTACTGCGAGTG3'; forward: 3'GCAGAAGGTGGAGCATGTTT5'; amplicon length: 491 bp). PCR-reactions were carried out in a thermal cycler using 1 µL DNA, 4.36 µL water, 0.06 Dream Taq (Thermo Scientific), 0.9 µL Buffer, 0.9 µL of each primer, and 0.9 µL dNTPs, and the following conditions: 95°C for 3 min, 35 cycles of 30 s at 95°C, 30 sec at 58°C for annealing, 1 min of extension at 72°C, ending with 20 min at 72°C. Forty-eight samples presented multiple bands, not correlated with mouth asymmetry. The desired fragment was isolated and extracted from agarose gel using the Zymoclean Gel DNA Recovery Kit (Zymo Research). 168 successfully amplified PCR products were post-processed and sequenced on a 3130xl ABI sequencer. The sequencer output was visualized and edited in FinchTV v. 1.5.0 (Geospiza, and then aligned using the ClustalW algorithm and default settings in MEGA v. 7.0 (Kumar *et al.*, 2016). The final alignment (190 bp) contained 106 variant positions, including heterozygous and uncertainties. The consensus sequence of the locus 56537 was incorporated in the alignment to localize the candidate SNP (56537-113), and each individual was genotyped at this position, which did not present any uncertainty.

Model selection approach to identify the best quantitative genetic model for the SNP 56537-113. The SNP genotype was used as dependent variable and coded under three different quantitative genetics models: totally additive (AA=0, AG/GA=0.5, GG=1), A dominant (AA=0, AG/GA=0, GG=1), and G dominant (AA=0, AG/GA=1, GG=1). Since we observed homozygous adult individuals for both alleles (SI 1), the model R (G) dominant and RR (GG) lethal (Hori *et al.*, 2007) was not included. To determine which model best characterizes this SNP, we fit these three models to mouth-bending angle using linear regression in R. The model with the lowest Akaike (AICc corrected for finite sample size; Akaike, 1973; Anderson, 2001; Hurvich and Tsai, 1989; Sakamoto *et al.*, 1986; Sugiura, 1978; *AICctab* function, library *bbmle* Bolker, 2016) and Bayesian information criterion (Sakamoto *et al.*, 1986; Schwarz, 1978; BIC functions in R) values was considered the mode of our SNP.

Tissue samples processing for stable isotopes analysis. All the soft tissues were removed from excised bones with a razor blade under a stereomicroscope to avoid contamination of the bone samples from other surrounding tissues having a different stable isotope value. All samples were dried at 50°C until there was no more change in mass and then manually pulverized. To account for the potential biasing effects of lipids within tissues (DeNiro and Epstein, 1978; Focken and Becker, 1998; Rolff and Elmgren, 2000), we chemically (Bligh and Dyer, 1959; Folch *et al.*, 1957) removed lipids from all samples. Lipids were extracted with three washes in a chloroform/methanol (2:1) solution and a final rinse with Milli-Q-Water. Lipid-purified samples were dried at 50°C for 72 hours and subsequently weighed. 0.9-1.3 mg of each sample was placed in tin capsules for analyses. Gas chromatography combustion isotope ratio mass spectrometry (GC-C-IRMS) was performed at the Isotopes Laboratory of the Limnological Institute of the University of Konstanz to determine $^{13}C/^{12}C$ and $^{15}N/^{14}N$ ratios. Measurements are reported in terms of delta values (δ) in permil (‰), i.e., parts per thousand differences relative to the standards (Pee Dee Belamnite limestone for C; nitrogen gas in the atmosphere for N).

Table S2. Specimens (individual IDs), mouth-bending angle, sampling location and SNP genotype data used for the SNP 56537-113 analysis.

Sample ID	Mouth bending angle	Mouth morph (R=right, L= left)	SNP 56537-113 genotype	Sampling location
AMCK10682	-1,89	R	GG	Mbita
AMCK10698	-3.48	R	GG	Mbita
AMCK10699	10.03	L	AA	Mbita
AMCK10702	-12.96	R	AG/GA	Mbita
AMCK10707	7.79	L	AG/GA	Mbita
AMCK10708	8.12	L	AA	Mbita
AMCK10709	-5.43	R	GG	Mbita
AMCK10710	-5.75	R	GG	Mbita
AMCK10711	2.36	L	AA	Mbita
AMCK10714	9.19	L	GG	Mbita
AMCK10718	-8.26	R	AG/GA	Mbita
AMCK10719	5.86	L	GG	Mbita
AMCK10722	-12.19	R	GG	Mbita
AMCK10724	-3.17	R	GG	Mbita
AMCK10725	-6.96	R	AA	Mbita
AMCK10729	-2.63	R	AG/GA	Mbita
AMCK10730	-5.81	R	AG/GA	Mbita
AMCK10732	-5.37	R	AG/GA	Mbita
AMCK10733	-7.44	R	AG/GA	Mbita
AMCK10734	-2.96	R	AA	Mbita
AMCK10735	-3.88	R	GG	Mbita
AMCK10736	-1.70	R	GG	Mbita
AMCK10737	6.07	L	AA	Mbita
AMCK10738	2.54	L	AG/GA	Mbita
AMCK10741	0.92	L	AA	Mbita
AMCK10742	-4.87	R	AA	Mbita
AMCK10743	-5.97	R	AG/GA	Mbita
AMCK10744	-10.89	R	GG	Mbita
AMCK10746	-1.63	R	GG	Mbita
AMCK10747	2.45	L	AA	Mbita
AMCK10748	-0.68	R	GG	Mbita
AMCK10749	-10.42	R	GG	Mbita
AMCK10750	-4.56	R	AG/GA	Mbita
AMCK10751	-2.17	R	AA	Mbita
AMCK10778	-6.78	R	AG/GA	Kasakalawe
AMCK10779	9.21	L	AG/GA	Kasakalawe

AMCK10780	-1.12	R	AA	Kasakalawe
AMCK10781	-16.82	R	GG	Katoto
AMCK10782	8.24	L	GG	Katoto
AMCK10783	3.16	L	AA	Katoto
AMCK10786	-3.14	R	GG	Katoto
AMCK10787	2.62	L	AA	Katoto
AMCK10788	-4.12	R	GG	Katoto
AMCK10789	3.93	L	AG/GA	Katoto
AMCK10790	8.31	L	AG/GA	Katoto
AMCK10791	0.34	L	GG	Katoto
AMCK10792	3.71	L	GG	Katoto
AMCK10794	3.44	L	AA	Katoto
AMCK10796	1.12	L	AG/GA	Katoto
AMCK10797	-1.27	R	AA	Katoto
AMCK10800	-5.37	R	AG/GA	Katoto
AMCK10801	-1.75	R	AA	Katoto
AMCK10802	8.30	L	AA	Katoto
AMCK10803	-6.40	R	AG/GA	Katoto
AMCK10805	1.44	L	AG/GA	Katoto
AMCK10806	-4.05	R	AA	Katoto
AMCK10807	-15.61	R	GG	Katoto
AMCK10808	-6.24	R	GG	Katoto
AMCK10810	15.04	L	AA	Katoto
AMCK10814	-9.08	R	AG/GA	Katoto
AMCK10815	5.90	L	AA	Katoto
AMCK10816	-12.56	R	GG	Katoto
AMCK10817	-17.72	R	AA	Katoto
AMCK10819	11.93	L	AG/GA	Katoto
AMCK10823	-6.22	R	AA	Katoto
AMCK10824	-18.60	R	AG/GA	Katoto
AMCK10825	-11.33	R	AG/GA	Katoto
AMCK10826	-11.88	R	AG/GA	Katoto
AMCK10828	7.45	L	AG/GA	Katoto
AMCK10829	5.72	L	AA	Katoto
AMCK10830	2.85	L	AG/GA	Katoto
AMCK10832	-15.26	R	GG	Katoto
AMCK10833	-7.28	R	AA	Katoto
AMCK10834	-14.07	R	GG	Katoto
AMCK10835	-0.98	R	GG	Katoto
AMCK10836	-3.62	R	GG	Katoto
AMCK10838	-0.92	R	AA	Katoto
AMCK10839	1.91	L	GG	Katoto
AMCK10841	-0.61	R	GG	Katoto
AMCK10843	-4.65	R	AG/GA	Kasakalawe

AMCK10844	-20.93	R	AG/GA	Kasakalawe
AMCK10846	0.67	L	AA	Kasakalawe
AMCK10847	-12.08	R	GG	Kasakalawe
AMCK10848	-13.93	R	GG	Kasakalawe
AMCK10849	-3.59	R	GG	Kasakalawe
AMCK10850	5.75	L	AA	Kasakalawe
AMCK10851	-4.81	R	AA	Kasakalawe
AMCK10852	-3.67	R	GG	Kasakalawe
AMCK10853	-5.01	R	GG	Kasakalawe
AMCK10855	-18.55	R	AG/GA	Kasakalawe
AMCK10856	-8.17	R	GG	Kasakalawe
AMCK10857	15.53	L	AA	Kasakalawe
AMCK10858	-8.16	R	AA	Kasakalawe
AMCK10859	-7.06	R	GG	Kasakalawe
AMCK10860	7.97	L	GG	Kasakalawe
AMCK10861	-4.51	R	GG	Kasakalawe
AMCK10862	-14.87	R	GG	Kasakalawe
AMCK10865	-16.91	R	GG	Kasakalawe
AMCK10866	-11.65	R	AG/GA	Kasakalawe
AMCK10868	-6.30	R	AA	Kasakalawe
AMCK10869	2.83	L	AG/GA	Kasakalawe
AMCK10870	-10.01	R	GG	Kasakalawe
AMCK10871	-8.52	R	AG/GA	Kasakalawe
AMCK10872	4.74	L	AA	Kasakalawe
AMCK10873	-6.95	R	AA	Kasakalawe
AMCK10874	12.42	L	AG/GA	Kasakalawe
AMCK10875	18.96	L	AA	Kasakalawe
AMCK10877	-0.89	R	AA	Kasakalawe
AMCK10878	-9.12	R	AA	Kasakalawe
AMCK10879	-13.15	R	GG	Kasakalawe
AMCK10880	-11.80	R	GG	Kasakalawe
AMCK10881	-3.07	R	AG/GA	Kasakalawe
AMCK10882	-2.96	R	AG/GA	Kasakalawe
AMCK10883	-1.45	R	GG	Kasakalawe
AMCK10885	-0.56	R	AG/GA	Kasakalawe
AMCK10886	7.11	L	GG	Kasakalawe
AMCK10887	4.64	L	AA	Kasakalawe
AMCK10888	2.75	L	GG	Kasakalawe
AMCK10889	-3.35	R	GG	Kasakalawe
AMCK10913	-8.41	R	GG	Toby
AMCK10914	-24.89	R	GG	Toby
AMCK10915	-10.18	R	AG/GA	Toby
AMCK10917	-18.17	R	AG/GA	Toby
AMCK10918	9.03	L	GG	Toby

AMCK10919	1.04	L	AG/GA	Toby
AMCK10920	-4.19	R	AA	Toby
AMCK10924	0.13	L	GG	Toby
AMCK10927	-2.43	R	GG	Toby
AMCK10928	-12.47	R	GG	Toby
AMCK10929	1.13	L	GG	Toby
AMCK10930	16.11	L	AA	Toby
AMCK10931	6.02	L	GG	Toby
AMCK10933	10.40	L	AG/GA	Toby
AMCK10935	11.67	L	GG	Toby
AMCK10936	-9.96	R	AA	Toby
AMCK10937	-3.61	R	GG	Toby
AMCK10938	3.31	L	GG	Toby
AMCK10961	5.25	L	AA	Toby
AMCK10962	5.12	L	GG	Toby
AMCK10966	-0.35	R	AG/GA	Toby
AMCK10968	-13.10	R	GG	Toby
AMCK10985	6.22	L	GG	Toby
AMCK10986	-3.02	R	GG	Toby
AMCK10989	2.84	L	GG	Toby
AMCK10990	-3.16	R	GG	Toby
AMCK10992	-4.88	R	GG	Toby
AMCK10995	-12.09	R	GG	Toby
AMCK11001	18.61	L	GG	Toby
AMCK11002	8.10	L	GG	Toby
AMCK11003	-17.47	R	GG	Toby
AMCK11005	9.27	L	AA	Toby
AMCK11012	5.16	L	GG	Toby
AMCK11013	6.84	L	GG	Toby
AMCK11015	-2.83	R	GG	Toby
AMCK11016	-5.48	R	AA	Toby
AMCK11017	-0.98	R	GG	Toby
AMCK11020	6.07	L	GG	Toby
AMCK11023	-9.80	R	GG	Toby
AMCK11027	-9.58	R	GG	Toby
AMCK11028	-6.27	R	GG	Toby
AMCK11031	-4.61	R	GG	Toby
AMCK11032	-4.62	R	GG	Toby
AMCK11035	-4.34	R	GG	Toby
AMCK11036	-11.11	R	GG	Toby
AMCK11037	-8.67	R	GG	Toby
AMCK11039	-4.54	R	GG	Toby
AMCK11040	2.97	L	GG	Toby
AMCK11044	-1.22	R	AA	Toby

Table S3. Specimens (individual IDs), mouth-bending angle, sampling location, body size, and stable isotopes information included in the stable isotopes study. Outliers that were excluded from analyses are highlighted in boldface. Abbreviations: S = symmetric, A= asymmetric. *values in this column are all negative; only the absolute values have been reported.

Sample ID	Mouth phenotype	Mouth bending angle	Sampling location	Average centroid size	Muscle $\delta^{15}N$	Muscle $\delta^{13}C$*	Bone $\delta^{15}N$	Bone $\delta^{13}C$*
AMCK10699	A	10.03	Mbita	10.81677111	7.796	20.867	6.305	19.663
AMCK10700	S	1.42	Mbita	14.79363818	8.683	16.054	6.298	13.469
AMCK10714	A	9.19	Mbita	12.8014612	7.553	17.965	6.118	16.828
AMCK10717	A	-13.09	Mbita	11.53236683	7.839	18.123	6.325	15.527
AMCK10740	A	12.01	Mbita	14.04313919	8.484	17.206	5.981	15.105
AMCK10741	S	0.92	Mbita	13.77198964	8.286	15.991	6.419	13.996
AMCK10748	S	-0.68	Mbita	10.98634659	7.143	19.990	6.293	18.585
AMCK10777	A	12.93	Kasakalawe	9.165493191	7.777	19.999	6.361	17.639
AMCK10779	A	9.21	Kasakalawe	10.93417346	8.513	15.665	6.637	12.742
AMCK10780	S	-1.12	Kasakalawe	9.334101214	8.170	19.192	6.575	17.503
**AMCK10781**	**A**	**-16.82**	**Katoto**	**14.0075323**	**10.221**	**16.196**	**7.805**	**13.828**
AMCK10791	S	0.34	Katoto	12.65950391	7.252	19.028	6.069	16.926
AMCK10796	S	1.12	Katoto	13.93834791	8.711	11.999	6.809	10.738
AMCK10797	S	-1.27	Katoto	15.69645427	8.621	17.274	7.060	15.251
AMCK10798	S	0.24	Katoto	15.33071349	8.913	14.103	7.044	11.709
AMCK10805	S	1.44	Katoto	13.45954688	8.960	14.928	7.483	12.556
AMCK10807	A	-15.61	Katoto	11.58906914	7.663	20.200	6.316	17.975
AMCK10810	A	15.04	Katoto	13.7342697	8.440	17.011	6.000	14.804
AMCK10813	S	1.29	Katoto	16.11541726	8.893	12.218	7.111	10.999
AMCK10817	A	-17.72	Katoto	13.37738084	8.728	16.581	7.038	15.075
AMCK10819	A	11.93	Katoto	12.72642402	8.250	18.754	6.736	16.144
AMCK10822	A	-13.26	Katoto	12.36131525	8.378	15.031	6.592	12.718
AMCK10824	A	-18.60	Katoto	11.90733166	7.974	17.560	6.668	15.352
AMCK10827	S	-0.93	Katoto	11.89284867	7.065	20.942	6.880	18.635
AMCK10831	A	8.85	Katoto	12.19153016	7.698	20.344	6.249	17.599
AMCK10832	A	-15.26	Katoto	11.94316676	8.113	17.308	7.195	14.785
AMCK10834	A	-14.07	Katoto	11.25160612	8.100	18.976	7.086	15.986
AMCK10835	S	-0.98	Katoto	11.56674769	8.988	16.683	7.680	14.344
AMCK10838	S	-0.92	Katoto	10.61525758	7.494	20.561	6.719	17.766
AMCK10840	S	-0.14	Katoto	10.48862642	8.020	17.956	6.622	15.197
AMCK10841	S	-0.61	Katoto	10.5088266	8.454	13.042	6.864	10.716
AMCK10844	A	-20.93	Kasakalawe	11.86954049	8.602	17.376	7.704	13.985
AMCK10846	S	0.67	Kasakalawe	10.90796704	8.213	18.344	7.460	14.909

AMCK10848	A	-13.93	Kasakalawe	10.67708375	8.416	18.653	6.840	15.796
AMCK10855	A	-18.55	Kasakalawe	10.20273733	7.692	18.809	6.471	15.596
AMCK10857	A	15.53	Kasakalawe	9.036176835	8.441	18.380	7.406	15.552
**AMCK10862**	**A**	**-14.87**	**Kasakalawe**	**12.4672682**	**11.701**	**13.970**	**10.143**	**11.181**
AMCK10865	A	-16.91	Kasakalawe	10.45247704	8.446	19.365	7.115	17.270
AMCK10874	A	12.42	Kasakalawe	10.96535102	8.390	19.918	6.785	17.740
AMCK10875	A	18.96	Kasakalawe	10.73852407	8.341	17.812	7.436	15.042
**AMCK10877**	**S**	**-0.89**	**Kasakalawe**	**10.3779274**	**9.945**	**16.410**	**8.772**	**14.173**
**AMCK10879**	**A**	**-13.15**	**Kasakalawe**	**12.0438074**	**10.964**	**15.120**	**9.885**	**12.695**
AMCK10883	S	-1.45	Kasakalawe	9.495661702	7.869	19.652	6.408	17.380
AMCK10884	S	1.70	Kasakalawe	10.03641775	8.148	19.866	6.901	17.808
AMCK10885	S	-0.56	Kasakalawe	10.55899669	8.902	19.803	6.765	17.408
AMCK10914	A	-24.89	Toby	16.34139578	9.379	12.380	6.681	9.696
AMCK10917	A	-18.17	Toby	10.90074368	7.741	18.896	6.857	17.123
AMCK10918	A	9.03	Toby	14.89919975	8.848	14.948	7.366	12.976
AMCK10919	S	1.04	Toby	13.35181338	9.073	15.933	7.032	13.483
AMCK10924	S	0.13	Toby	13.75319093	8.688	15.468	7.194	12.974
AMCK10925	S	-1.01	Toby	13.24307438	8.527	15.106	7.116	12.791
AMCK10929	S	1.13	Toby	11.28472806	8.584	17.399	7.201	14.237
AMCK10930	A	16.11	Toby	11.68175842	8.179	16.040	6.940	15.104
AMCK10932	S	1.65	Toby	11.29544937	7.231	18.861	6.029	16.645
AMCK10933	A	10.40	Toby	11.4861538	7.645	17.402	6.792	15.123
AMCK10935	A	11.67	Toby	11.52045516	8.611	17.362	6.590	14.793
AMCK10957	S	-0.73	Toby	14.47769618	8.796	11.937	7.463	9.373
AMCK10958	S	0.08	Toby	11.2959027	7.807	14.640	6.553	11.535
AMCK10966	S	-0.35	Toby	14.65671435	8.977	15.191	7.674	13.183
AMCK10994	S	0.35	Toby	13.4305561	8.775	12.878	7.226	11.258
AMCK11001	A	18.61	Toby	13.4759738	8.079	13.569	6.463	11.187
AMCK11003	A	-17.47	Toby	15.97929973	8.872	14.416	7.773	11.330
AMCK11005	A	9.27	Toby	14.92678624	8.826	12.715	7.702	10.460
AMCK11017	S	-0.98	Toby	17.60453704	8.931	13.160	7.349	10.895
AMCK11019	S	1.01	Toby	16.04025969	9.145	12.334	7.278	9.620
AMCK11021	S	-0.95	Toby	16.41693882	8.770	11.471	7.072	9.358
AMCK11029	S	0.66	Toby	13.97903332	9.475	13.228	7.195	11.474
AMCK11044	S	-1.22	Toby	13.36113532	8.591	13.536	7.596	11.789

Table S4. The best quantitative genetic model describing the relationship between the mouth bending angle and SNP 56537-113 according to the AICc and BIC results. Abbreviations: AICc=Akaike information criterion corrected for finite sample size; BIC=Bayesian information criterion; df=degree of freedom.

Model	df	AICc	BIC
G dominant	3	1180.4	1189.590
Additive	3	1182.3	1191.505
A dominant	3	1186.6	1195.804

Figure S5. Scatter plot of muscle carbon and nitrogen isotopic values. Ellipses represent the groups (phenotypes) confidence intervals (95%). Colors and shapes correspond to mouth phenotypes (blue triangles identify symmetric samples; orange circles denote asymmetric individuals).

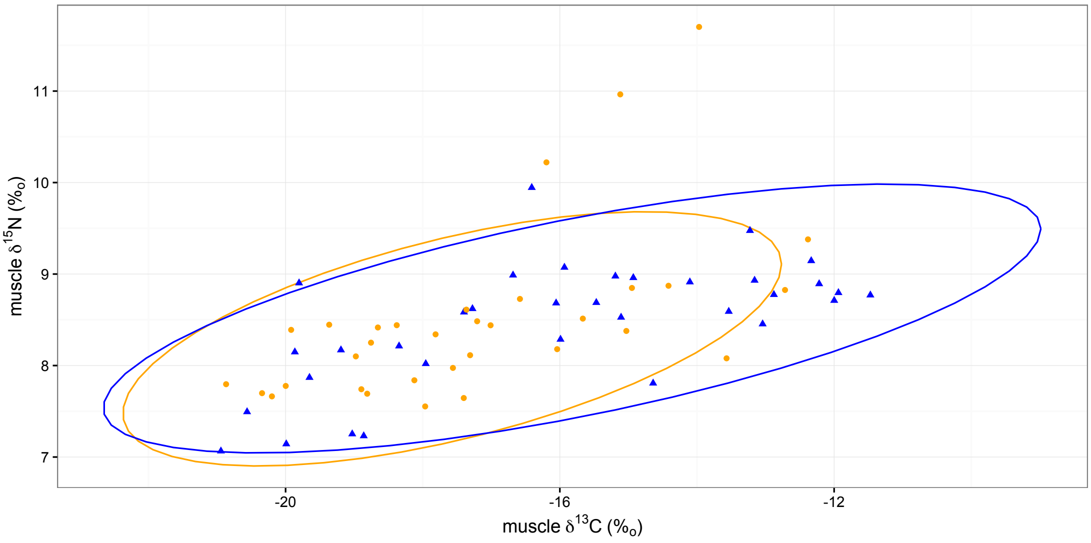

Figure S6. Scatter plot of bone carbon and nitrogen isotopic values. Ellipses represent the groups (phenotypes) confidence intervals (95%). Colors and shapes correspond to mouth phenotypes (blue triangles identify symmetric samples; orange circles denote asymmetric individuals).

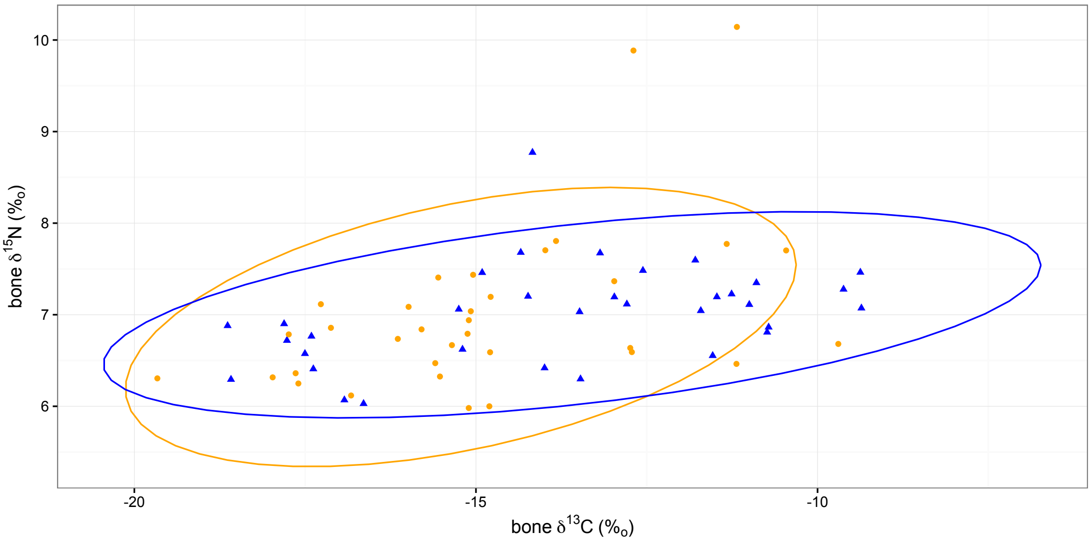

Table S7. Model selection for the stable isotope analyses. Three outliers were excluded from both the muscle and bone datasets (see main text). Abbreviations: LRT=Likelihood ratio test; AICc=Akaike information criterion corrected for finite sample size; df=degree of freedom. Significant P-value (< 0.05) is reported in boldface.

Tissue	Stable isotope	Variable(s) included in the model	LRT					AICc	
			Res.Df	RSS	Df	Sum of Sq.	Pr (>Chi)	AICc	df
Muscle	$\delta^{13}C$	Mouth phenotype	63	420	-	-	-	312.1	3
		Mouth phenotype + location	60	279.4	3	140.63	**$8.179e^{-11}$**	292.7	6
		Mouth phenotype + location + size	59	166.1	1	113.30	**$2.240e^{-10}$**	261.4	7
	$\delta^{15}N$	Mouth phenotype	63	21	-	-	-	117.4	3
		Mouth phenotype + location	60	18.63	3	2.3745	**0.008**	116.7	6
		Mouth phenotype + location + size	59	11.85	1	6.7746	**$6.374e^{-9}$**	89.8	7
Bone	$\delta^{13}C$	Mouth phenotype	63	425	-	-	-	312.9	3
		Mouth phenotype + location	60	287.2	3	137.77	**$3.331e^{-10}$**	294.5	6
		Mouth phenotype + location + size	59	172.6	1	114.62	**$3.877e^{-10}$**	263.9	7
	$\delta^{15}N$	Mouth phenotype	63	14.92	-	-	-	95.2	3
		Mouth phenotype + location	60	11.21	3	3.7062	**$5.819e^{-05}$**	83.7	6
		Mouth phenotype + location + size	59	9.833	1	1.3786	**0.004**	95.2	7

Table S8. Model selection for the stable isotope analyses using the constant or structured variance (*gls* function). Three outliers were excluded from both the muscle and bone datasets (see main text). Abbreviations: AIC=Akaike information criterion; BIC=Bayesian information criterion; logLik=log lkelihood; L.Ratio=likelihood ratio; AICc=Akaike information criterion corrected for finite sample size; df=degree of freedom; NVS=no variance structure, constant variance model; VP=variance depend on phenotypes, structured variance model. Significant P-value (< 0.05) is reported in boldface.*values in this column are all negative; only the absolute values have been reported.

Tissue	Stable isotope	Model	anova					AICc	
			AIC	BIC	logLik*	L.Ratio	P-value	AICc	df
Muscle	$\delta^{13}C$	NVS	261.11	275.65	123.55	-	-	263.1	7
		VP	257.89	274.51	120.95	5.2175	**0.0224**	260.5	8
	$\delta^{15}N$	NVS	105.36	119.90	45.678	-	-	107.3	7
		VP	95.639	112.26	39.819	11.718	**6e^{-04}**	98.2	8
Bone	$\delta^{13}C$	NVS	263.37	277.91	124.68	-	-	265.3	7
		VP	262.02	278.64	123.01	3.3502	0.0672	264.6	8
	$\delta^{15}N$	NVS	94.312	108.85	40.156	-	-	98.0	7
		VP	95.418	112.04	39.709	0.8942	0.3444	96.3	8

Table S9. MANCOVA results of the stable isotope dataset. Three outliers were excluded from both the muscle and bone datasets (see main text). Abbreviations: df=degree of freedom. Significant P-value (< 0.05) is reported in boldface.

Tissue	Variable	Pillai	Df num, df den	Approximate F-value	P-value
Muscle	Mouth phenotype	0.15809	2,58	5.4455	**0.006803**
	Location	0.59733	6,118	8.3752	**$1.433e^{-07}$**
	Size	0.45986	2,58	24.6902	**$1.748e^{-08}$**
Bone	Mouth phenotype	0.13278	2,58	4.4402	**0.01606**
	Location	0.63470	6,118	9.1426	**$3.268e^{-08}$**
	Size	0.41361	2,58	20.4552	**$1.894e^{-07}$**

Table S10. Univariate PERMANOVA results of the stable isotope dataset. Three outliers were excluded from both the muscle and bone datasets (see main text). Abbreviations: df=degree of freedom. Significant P-value (< 0.05) is reported in boldface.

Tissue	Stable isotope	Variable	Df num, df den	F model	R-squared	P-value
Muscle	$\delta^{13}C$	Mouth phenotype	1, 59	10.855	0.06782	**0.003**
		Location	3, 59	16.651	0.31210	**0.001**
		Size	1, 59	40.245	0.25145	**0.001**
	$\delta^{15}N$	Mouth phenotype	1, 59	3.958	0.03648	0.064
		Location	3, 59	3.939	0.10893	**0.024**
		Size	1, 59	33.717	0.31078	**0.001**
Bone	$\delta^{13}C$	Mouth phenotype	1, 59	8.592	0.05585	**0.007**
		Location	3, 59	15.696	0.30605	**0.001**
		Size	1, 59	39.174	0.25462	**0.001**
	$\delta^{15}N$	Mouth phenotype	1, 59	1.6414	0.01801	0.224
		Location	3, 59	7.4128	0.24397	**0.001**
		Size	1, 59	8.2716	0.09075	**0.008**

Table S11. Multivariate PERMANOVA results of the stable isotope dataset. Three outliers were excluded from both the muscle and bone datasets (see main text). Abbreviations: df=degree of freedom. Significant P-value (< 0.05) is reported in boldface.

Tissue	Variable	Df num, Df den	F model	R-squared	P-value
Muscle	Mouth phenotype	1, 59	10.395	0.06637	**0.002**
	Location	3, 59	15.804	0.30272	**0.001**
	Size	1, 59	39.811	0.25419	**0.001**
Bone	Mouth phenotype	1, 59	8.217	0.05461	**0.005**
	Location	3, 59	15.249	0.30403	**0.001**
	Size	1, 59	37.508	0.24927	**0.001**

Table S12. Results of the stable isotope analyses using the structured variance (variance depending on phenotypes, VP models). Three outliers were excluded from both the muscle and bone datasets (see main text). Abbreviations: df=degree of freedom. Significant P-value (< 0.05) is reported in boldface.

Tissue	Stable isotope	Variable	Df num, df den	F-value	P-value
Muscle	$\delta^{13}C$	Mouth phenotype	1, 59	11.344	**0.0013**
		Location	3, 59	18.603	**<0.0001**
		Size	1, 59	45.427	**<0.0001**
	$\delta^{15}N$	Mouth phenotype	1, 59	4.24	**0.0440**
		Location	3, 59	5.45	**0.0022**
		Size	1, 59	47.07	**<0.0001**
Bone	$\delta^{13}C$	Mouth phenotype	1, 59	8.687	**0.0046**
		Location	3, 59	15.978	**<0.0001**
		Size	1, 59	44.811	**<0.0001**
	$\delta^{15}N$	Mouth phenotype	1, 59	1.635	0.2060
		Location	3, 59	7.448	**0.0003**
		Size	1, 59	8.683	**0.0046**

Supplementary References

Akaike, H. 1973. Maximum likelihood identification of Gaussian autoregressive moving average models. Biometrika, 255-265.

Altschul, S. F., Madden, T. L., Schäffer, A. A., Zhang, J., Zhang, Z., Miller, W. & Lipman, D. J. 1997. Gapped BLAST and PSI-BLAST: a new generation of protein database search programs. Nucleic acids research, 25, 3389-3402.

Anderson, M. J. 2001. A new method for non-parametric multivariate analysis of variance. Austral ecology, 26, 32-46.

Bligh, E. G. & Dyer, W. J. 1959. A rapid method of total lipid extraction and purification. Canadian journal of biochemistry and physiology, 37, 911-917.

Bolker, B. 2016. R Development Core Team, 2014. bbmle: Tools for General Maximum Likelihood Estimation. R package version 1.0. 16. Computer program.

Brawand, D., Wagner, C. E., Li, Y. I., Malinsky, M., Keller, I., Fan, S., Simakov, O., Ng, A. Y., Lim, Z. W. & Bezault, E. 2014. The genomic substrate for adaptive radiation in African cichlid fish. Nature, 513, 375-381.

DeNiro, M. J. & Epstein, S. 1978. Influence of diet on the distribution of carbon isotopes in animals. Geochimica et cosmochimica acta, 42, 495-506.

Focken, U. & Becker, K. 1998. Metabolic fractionation of stable carbon isotopes: implications of different proximate compositions for studies of the aquatic food webs using δ13C data. Oecologia, 115, 337-343.

Folch, J., Lees, M. & Sloane-Stanley, G. 1957. A simple method for the isolation and purification of total lipids from animal tissues. J biol Chem, 226, 497-509.

Fruciano, C. 2016. Measurement error in geometric morphometrics. Development genes and evolution, 226, 139-158.

Geospiza 2009. FinchTV. Washington.

Hori, M., Ochi, H. & Kohda, M. 2007. Inheritance pattern of lateral dimorphism in two cichlids (a scale eater, Perissodus microlepis, and an herbivore, Neolamprologus moorii) in Lake Tanganyika. Zoological Science, 24, 486-492.

Hurvich, C. M. & Tsai, C.-L. 1989. Regression and time series model selection in small samples. Biometrika, 297-307.

Kumar, S., Stecher, G. & Tamura, K. 2016. MEGA7: Molecular Evolutionary Genetics Analysis version 7.0 for bigger datasets. Molecular biology and evolution, msw054.

Petersen, C. G. J. 1894. On the biology of our flat-fishes and on the decrease of our flat-fish fisheries, with some observations showing how to remedy the latter and promote the flat-fish fisheries in our seas east of the Skaw, Kjøbenhavn Centraltrykkerie.

Raffini, F., Fruciano, C., Franchini, P. & Meyer, A. 2017. Towards understanding the genetic basis of mouth asymmetry in the scale-eating cichlid Perissodus microlepis. Molecular ecology, 26, 77-91.

Raffini, F., Fruciano, C. & Meyer, A. in press. Morphological and genetic correlates in the left-right asymmetric scale-eating cichlid fish of Lake Tanganyika Biological Journal of the Linnean Society.

Rolff, C. & Elmgren, R. 2000. Use of riverine organic matter in plankton food webs of the Baltic Sea. Marine Ecology Progress Series, 197, 81-101.

Sakamoto, Y., Ishiguro, M. & Kitagawa, G. 1986. Akaike information criterion statistics, Dordrecht, The Netherlands, D. Reidel.

Schwarz, G. 1978. Estimating the dimension of a model. The annals of statistics, 6, 461-464.

Sugiura, N. 1978. Further analysis of the data by Akaike's information criterion and the finite corrections. Communications in Statistics-Theory and Methods, 7, 13-26.

Takeuchi, Y., Hori, M., Tada, S. & Oda, Y. 2016. Acquisition of Lateralized Predation Behavior Associated with Development of Mouth Asymmetry in a Lake Tanganyika Scale-Eating Cichlid Fish. PloS one, 11, e0147476.

Untergasser, A., Nijveen, H., Rao, X., Bisseling, T., Geurts, R. & Leunissen, J. A. 2007. Primer3Plus, an enhanced web interface to Primer3. Nucleic acids research, 35, W71-W74.
